# Evolutionary origins of temporal discounting: Modeling how time and uncertainty constrain optimal decision-making strategies across taxa

**DOI:** 10.1371/journal.pone.0310658

**Published:** 2024-11-12

**Authors:** Brian Villmoare, David Klein, Pierre Liénard, Timothy S. McHale

**Affiliations:** 1 Department of Anthropology, University of Nevada Las Vegas, Las Vegas, Nevada, United States of America; 2 University of California, San Luis Obispo, California, United States of America; 3 Social Sciences Department, California Polytechnic State University, San Luis Obispo, California, United States of America; RIKEN CBS: RIKEN Noshinkei Kagaku Kenkyu Center, JAPAN

## Abstract

The propensity of humans and non-human animals to discount future returns for short-term benefits is well established. This contrasts with the ability of organisms to unfold complex developmental sequences over months or years efficiently. Research has focused on various descriptive and predictive parameters of ‘temporal discounting’ in behavior, and researchers have proposed models to explain temporal preference in terms of fitness-maximizing outcomes. Still, the underlying ultimate cause of this phenomenon has not been deeply explored across taxa. Here, we propose an ultimate (i.e., evolutionary) causal explanation for the selection of temporal discounting largely conserved across taxa. We propose that preference for a short-term reward (e.g., heightened impulsivity) often is less than optimal and likely is the product of constraints imposed on natural selection with respect to predicting events in a temporal framework in the context of future uncertainty. Using a simple Newtonian model for time across a fitness landscape in which movement by organisms is only possible in one direction, we examine several factors that influence the ability of an organism to choose a distant reward over a more temporally proximate reward: including the temporal distance of the far reward, the relative value of the distant reward, and the effect of uncertainty about the value and presence of the distant reward. Our results indicate that an organism may choose a more distant reward, but only if it is not too far into the future and has a substantially higher-value fitness payoff relative to the short-term reward. Notably, any uncertainty about the distant reward made it *extremely unlikely* for an organism to choose the delayed reward strategy compared to choosing a closer reward, even if the distant reward had a much higher payoff because events that are uncertain are only partially visible to natural selection pressures. The results help explain why natural selection is constrained to promote more optimal behavioral strategies and why it has difficulty selecting a distant reward over a lower-value short-term reward. The degree of uncertainty is an especially salient ecological variable in promoting and preferencing short-term behavioral strategies across taxa. These results further help illustrate why, from an ultimate causal perspective, human and non-human taxa have difficulty making more optimal long-term decisions.

## Introduction

Time is a well-understood significant selective force in evolutionary developmental biology and ethology, both of which investigate ultimate and proximate causal mechanisms leading to anatomical and behavioral phenotypic variation [1, 2]. Organisms must operate in a temporal framework in which the regular progression of the temporal dimension must be dealt with for the organism to survive. The occurrence of events in time, both in terms of sequence and interval, can have significant implications for an individual organism. Developmental sequences that occur out of order or in too short or long a time may be subject to strong negative selection (i.e., the selective removal of alleles that are deleterious), as can behaviors that occur in temporally disadvantageous order or timeline.

Selection favors developmental and optimal economic behavioral strategies that, on average, result in the greatest fitness payoffs [3], which are significantly determined by the temporal dimensions of an organism’s ecological niche. One particularly noteworthy aspect of dealing with the temporal dimension is the fact that the results of present decisions necessarily occur in the future. Unlike the present and the past, which are open to direct examination, the future is unknown to a greater or lesser degree. This ‘unknown’ introduces an element of uncertainty that organisms must deal with.

Here, we address the relationship between time and unknown future risks and rewards that influence how organisms (specifically animals) deal with these parameters. The principles apply to any element of organismal biology that must appear in a specific sequence at a specific time, but we contrast two specific instances in which there appear to be strongly divergent patterns concerning time (development and behavior) and model an ultimate causal approach to explain the phenomena of temporal discounting. In doing so, we build and expand on previous work in the human and non-human animal literature, which model elicit risk and time preferences together to estimate impulsivity more accurately [e.g., 4–9]. We aim to provide a broader application of delay-discounting models that are likely conserved across taxa to aid evolutionary theorists in understanding the phylogenetic constraints and conditions that would preferentially select for short- versus long-term behavioral strategies. This model also has the potential to generate new testable hypotheses and predictions by assessing the parameters that would be required in a given ecological setting to promote psychological predispositions in an organism that would forgo an immediate reward (e.g., cost in terms of lack of energetic resources) and favor a long-term delayed reward strategy (e.g., benefit in terms of fitness by enhancing lifetime reproductive success).

### Time and timing

The concept of time is philosophically elusive, and there is no broadly accepted definition across disciplines [10]. Despite the difficulty of defining this concept, it is clear that the temporal occurrence of events, both in terms of sequence and relative to the passing of specific amounts of time, is of critical importance in phylogenetics, evolutionary biology, and the behavioral sciences. Biological events’ sequence and timing are subject to natural selection pressures [9, 11]. The lack of a consensus on how best to define time adds to the challenge of accurately modeling the effects of time in ways that fully account for all of its characteristics. The 2nd Law of Thermodynamics applies to any macroscopic system, and ‘time’s arrow’ prevents movement backward in the temporal dimension [12]. In non-relativistic (e.g., Newtonian) physics, time is typically modeled as a linear dimension, with the special property that movement can only occur in one direction. Much as the location of a thing or event can be described by a location in spatial dimensions, the occurrence of an event can be described with a location on the (single) temporal dimension [13, 14].

Because the linear-dimension model of time offers the fewest assumptions and provides an intuitively satisfying description of the sequential unfolding of events, we use such a model in this study. We treat time simply as a linear dimension along which events occur at particular locations on the axis, with the caveat that movement can occur only in one direction, and only the past and present can be directly observed. In our model, the distance between events is inferred to have potential significance for the effects of selection.

Critical to our analysis is the idea that, because of the unidirectional movement along the single dimension of time, as decisions are made in a temporal framework, there are costs and benefits associated with those decisions that cannot be avoided. One cannot simply ‘go around’ an event in time as one would an obstacle in space. In this view, each organism is imbued with a set of implicit biases influencing behavioral strategies that are best understood as the product of evolutionary trade-offs, designed to maximize one’s fitness when navigating an unpredictable world.

### Time and anatomical development

The timing of events in the lifetimes of organisms is critical, and various genetic mechanisms have evolved to track the passage of time at various scales [15–19]. Developmental sequences of a given species map out with regularity across time, and important changes often relate to changes in timing. *Homo sapiens*, for example, in comparison to other apes, are characterized by delayed rates of sexual maturation, long childhood dependency, and differential rates of growth (e.g., brain size, body size) and development (shape) across the lifespan [20, 21]. Other changes in timing and sequence (e.g., humans, uniquely among primates, acquire their canines early, roughly at the same time as their incisors–[22, 23] are likely the product of important adaptive transitions. In other words, organisms face different adaptive challenges at different life course stages [9]. As such, natural selection has generated considerable variation around this basic scheme in the animal kingdom to optimize fitness. Understanding the role of time in these developmental processes and the advantages and disadvantages for the organisms of shifts in timing is key to understanding the reasons for evolutionary changes.

### Time and behavior

The propensity of humans and non-human animals to discount future returns for short-term benefits is well-known in disciplines that focus on decision-making, such as Economics and Behavioral Sciences [e.g., 5, 7, 8, 24–29]. In Behavioral Economics, this phenomenon is known as ‘temporal discounting,’ ‘delay preference,’ or ‘time preference,’ and research has focused on various descriptive and predictive parameters, such as the shape of the discounting curve, conditions that might affect the length of the long-term decision window, and differences among social and political human groups, in various economic, age, and health circumstances [e.g., 28, 30–42].

Psychologists have explored the difficulty of making these decisions at length [e.g., 43]. Perhaps the most well-known example is the Stanford ‘marshmallow experiment’ [44]. Children were offered a single marshmallow and told they would receive another if they could sit and forestall eating that first marshmallow for 15 minutes. In this experiment, the children faced a decision they could not avoid, and whichever choice they made, they had to bear a cost. The cost of eating the first marshmallow was not receiving the second marshmallow, but, importantly, the decision to wait also imposed costs. Waiting meant not receiving the benefit of eating the first marshmallow in that 15-minute period (and the theoretical potential for it to be taken away). Most children were unable to bear that cost. Thus, the cost of waiting was a substantial barrier to the more distant but greater reward.

Some researchers have proposed models under which temporal discounting might be seen as a rational (i.e., economical) decision in the broader context of an animal’s lifetime [e.g., 29, 45–51]. Economists have explored this idea from a biological perspective [e.g., 51–58], although rarely from the perspective of ultimate causation [59]. However, it may also be true that temporal discounting is not the most optimal long-term strategy; rather, it may be a by-product of how natural selection acts in the temporal framework. We propose that for many behaviors, particularly those under the strong influence of natural selection, animals may be constrained from making the most efficient, long-term choice. Temporal discounting is, under our model, a by-product of the phylogenetic constraint imposed by the process of natural selection, similar to the limitations under which natural selection generates novel anatomical forms. We test this hypothesis by employing a model of change under natural selection over time, using a fitness-landscape algorithm [60].

### Factors influencing temporal discounting

Our model is quantitative, and several factors may affect the evolution of response to intertemporal choice.

### 1. Value of Future Returns

The adaptive importance of future events is framed in relation to the potential value of the event occurring in the present and the attendant costs of delay [29, 45, 48, 61–63]. The utility of teeth for primates is clear, but canine eruption is delayed because the appearance of adult canines in non-human primates acts as a trigger–other individuals of that species treat any individual with fully erupted canines as an adult [23, 64–66]. In males, this means entering competition with the fully developed males of the social group. Early canine eruption would provide potential benefits for a juvenile in terms of self-defense, but socially it might well spell the demise of a young male primate. Thus, the ‘future’ event is more valuable than the ‘present’ event when considering the net sum of the fitness reward versus the cost with respect to dental eruption timing in primates.

Behaviorally, the value of a future reward can be weighed against the value of a present reward. For example, the decision by an animal to cache food is an economic decision–what is the value of that food consumed in the present (or the investment in time and energy to acquire the food) balanced against the potential value of that food in the future, when resources may be scarce?

### 2. Reliability of Predictions

For future events to act as agents of selection, these events must reliably appear in the future. If a cicada delays adult development from the nymph stage for 17 years, the reproductive benefit of that delay (avoiding predation or swamping out predators) must actually hold. If a western scrub-jay (*Aphelocoma californica*) caches food [67], the reliability of the cache creates a strong selective force: if the scrub-jay caches the food but is never able to retrieve it (due to thievery by other animals, for example, or weather effects), the behavior is selected against. However, if the food can be reliably retrieved in the future, the behavior would be selected. However, reliability is not binary–the predictability of the future reward may follow a probability distribution that would be influenced by factors such as the length of time to the future reward and the general predictability of the parameter (e.g., weather, cache-robbing, etc.). Experimental studies have shown that in cognitively advanced animals such as apes, the reliability of future reward is a factor in temporal preference [68, 69]; wild animals will similarly alter behavior when they perceive a change in the probability of the reward [70].

### 3. Temporal Distance

Events do not simply happen in the past, present, or future; time is a scalar dimension. In particular, future events may occur in the near-term future or more distantly. The distance into the future of an event is likely to have multiple effects on the value of that event to an organism. One effect of greater temporal distance is likely on the reliability of the outcome. The further into the future an event will occur, the less predictable it is [71]. Past events are normally used to predict future conditions, but further into the distant future, the resolution degrades, and nonlinearity accumulates. Additionally, the further into the future an event occurs, the greater the potential cost of deferring (delay discounting). If a future event requires too much investment of resources over a longer time period, or if the cost of continual deferment is too great, the event may not be sufficiently beneficial to be selected for. This means that the temporal distance into the future of an event is interdependent with the other two parameters. In experimental conditions, the deferment time appears to be generally unrelated to the lifespans of the animals [47], underlining the importance of understanding the factors driving the scalar quantities of time in decision-making.

### Model—Part 1: The value of future returns

To model the adaptive response to future rewards of varying values, we use an evolutionary simulation to generate a pattern from which we derive a mathematical relationship. To do this, we expand upon concepts of modeling phenotypic evolution [‘meso’-evolutionary theory; 73, 74] from Adaptive Dynamics [5, 75–78] and Wright’s [72] idea of the general fitness landscape [60, 79].

**Fig 1:**
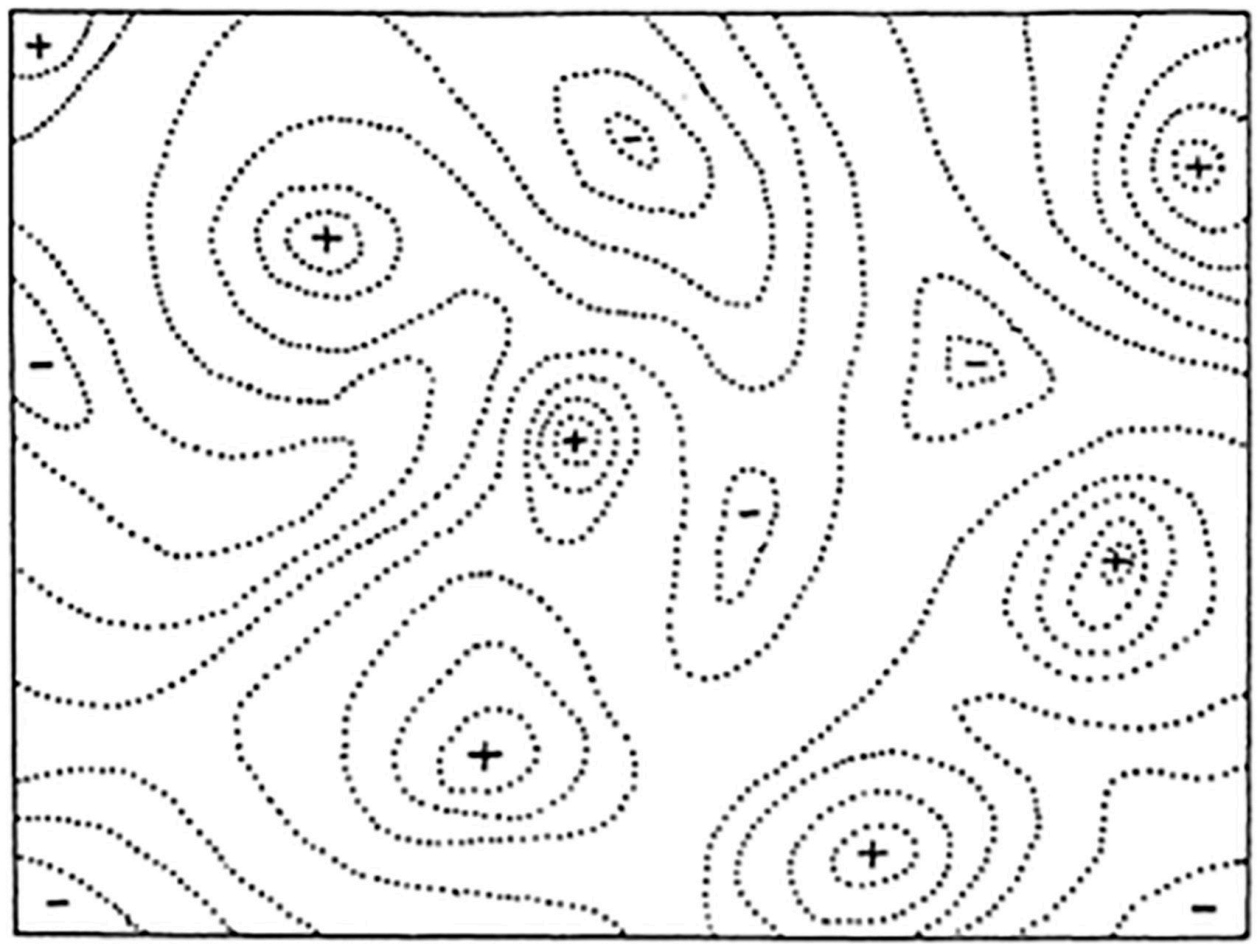
Sewell Wright’s original fitness landscape. Sewell Wright first proposed the use of a fitness landscape for visualizing the interactions of particular genetic combinations in 1932 [72]. His visualization (left) presented the fitness landscape as a two-dimensional model with continuous expression of the two genes along the respective axes. Under specific environmental conditions, for given organisms, particular combinations of genetic expression are adaptive, whereas others are maladaptive, and the adaptive landscape helps visualize this. Combinations of high adaptive value are expressed as high points, or peaks, whereas relatively maladaptive combinations are low spots, or valleys. The peaks and valleys are represented by + and -. Although the original visual model uses a two-dimensional landscape, Wright notes that the thousands of genes with their millions of potential combinations meant that organisms operated in an n-dimensional landscape. Wright points out that the peaks are in no way fixed and operate in an unseen temporal framework. Should the environment change over time, the peak might move (or disappear), such that a different combination of allele frequencies would constitutes a new peak, or optimum value.

We incorporate time as an axis on the fitness landscape, with specific reference to the multi-peak problem identified by Sewell Wright [72], under which populations on a low peak cannot move to a higher peak because of the cost of moving off the low peak (Fig 1). Such a redefined fitness landscape allows us to model the unfolding sequence of events that predictably shape selection for organismal events (developmental or behavioral) across time. Further, this perspective may offer new insight into how and under what conditions equilibrium for a particular trait(s) is met and under what conditions new phenotypes would be favored.

Our model applies to phenotypes, whether anatomical or behavioral. Note that our model follows Wilson [40], under which evolutionary theory is as effective at explaining arthropod behavior as it is at explaining human behavior. Here, there are greater or lesser optimal ‘decisions’ that result in differential reproductive success. Still, the organisms themselves need not ‘know’ that they are making the correct decision, even though they are ‘rewarded’ for the correct decisions. We observe that treating intelligence as an ‘emergent property’ at any scale implies that behavior must be treated equally at levels of high and low complexity [80]. We regard the behavioral model as (1) more consistent with this broader interpretation of decision-making, (2) most compatible with an evolutionary framework, and (3) not necessitating an ad-hoc decision of when to invoke cognitive models for non-human animal behaviors.

### Time as a dimension on the fitness landscape

Traditionally, the fitness landscape takes the expression of alleles as axes, with the peaks representing allelic combinations [72]. In our mathematical model, the phenotype (developmental or behavioral) is under selection pressure, and selection on the phenotype is modeled directly. We extend the model to factor in time. Under this formulation, as agents move through the fitness landscape, they also move through the temporal landscape so that specific locations on the fitness landscape are more temporally distant. In this fitness landscape, ‘choice’ cannot be avoided. Populations moving through the temporal landscape cannot avoid making the ‘decision’ to adopt one of the two peaks because moving to the more distant peak (with its greater reward) necessarily means moving over (and off) the near peak and thereby losing the rewards of that near peak. This model should permit the determination of the conditions under which populations of agents should move off lower but closer peaks to (temporally) distant and higher-value peaks. Note that, in our model, movement of the population is only permitted in one direction, mirroring the fact that, under Newtonian models of time-space, we can only move one direction along the temporal axis (unlike more traditional fitness landscape models).

### Methods—Part 1: The value of future returns

We assume a one-dimensional landscape in our model and note that our results can be extended to multiple dimensions if populations are constrained to move directly through the ‘decisions.’

**Fig 2:**
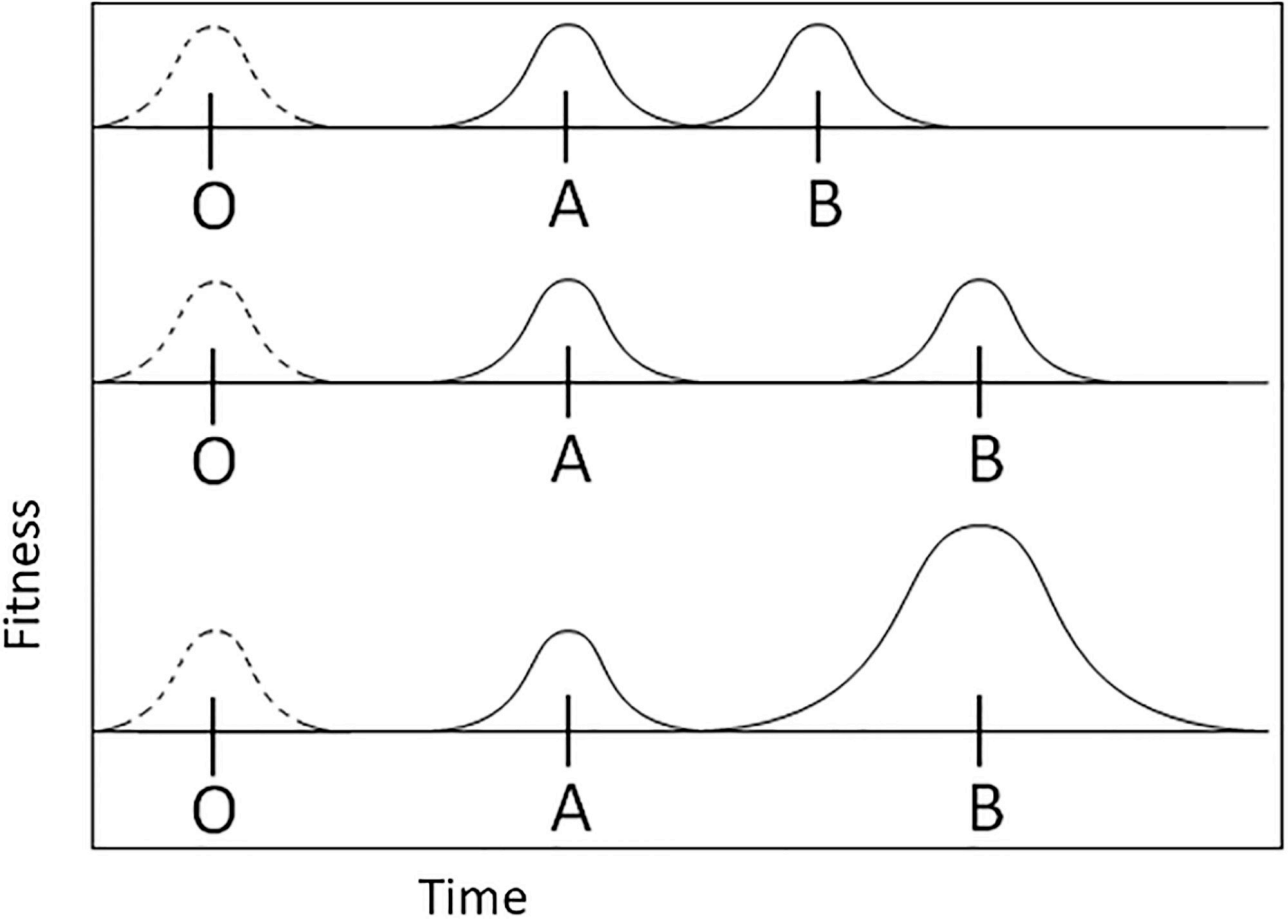
Simulated one-dimensional model of the fitness landscape. The origin (O) represents a peak that has disappeared. The population must then move to a new peak in the future (time is represented by distance along the X-axis). Peak A is fixed in fitness (expressed by height / vertical axis) and position relative to the origin (O). Peak B varies in position and strength, starting at the same location and strength as Peak A, then iteratively becoming more distant from the origin (O). At each greater distance, the height of the peak is increased until the strength of selection is sufficient to draw the population from A to B. In this figure, the likelihood of survival and reproduction for an agent is expressed as height on the y-axis.

We simulate a population of agents at equilibrium at an adaptive peak, which are faced with its disappearance (Fig 2, O), and the emergence of *two* other peaks (Fig 2A and 2B), one more distant than the other. We rely on a simple heuristic to model agents’ behavior. They follow a random-walk pattern, with reproduction and death events based on peak proximity. Agents can reproduce or die but do not move, so the population of agents moves by death and reproduction. Agents closer to a peak are more likely to survive and produce offspring so that the population as a whole will move across the fitness landscape toward a peak.

The agents do not know their location, only the relative desirability of their location (which is determined by the proximity and value [height] of a peak). To test the effects of temporal distance and distal reward value, the height and distance of the far peak are varied to determine the indifference point at which organisms ignore the short-term reward of the closer peak for the more distant reward of the further peak.

Assume the agents in our model follow a simple stochastic reproduction/survival heuristic based on location relative to a peak (and the height of that peak). The higher the ‘desirability’ of an agent’s location, the greater the probability that the agent will create offspring and survive to the next timestep in the simulation (survival and reproduction are different events). The descendants (clonal) of any one individual are placed via the assumption of a low mutation rate, with variation from the parental value based on a relatively narrow normal random distribution centered at the parental location.

The population (n = 500) starts at origin (O) and is subjected to probability-driven selection/reproduction based on proximity to Peaks A and B. The closer any individual is to one of the peaks (A or B), the higher the likelihood of survival and reproduction (although no individual has a non-zero probability). In the first iteration, the peaks are of equal strength, and at each iteration, the strength (e.g., ‘height’) of peak (B) is increased up to 20x the ‘height’ of peak (A) closer to the origin (Fig 2). Each iteration cycles through for 5,000 generations (each a cycle of selection and reproduction). In the simplest model used here, Peak B starts at the same distance from the origin (O) as Peak A, but the distance from O to B increases throughout the model’s run to evaluate the effect of increasing the distance of the far peak. The model thusly determines how high a particular peak would have to be at any given distance from the O to attract the population. We do not employ any direct feedback from the population to environmental parameters beyond carrying capacity (500). Reproductive rates are kept high enough to avoid extinction, although population numbers may decrease and increase under selective pressure, with an upper limit of 500 individuals expressing the environmental ‘carrying capacity’. Note that the selection forces act as a ratchet, precluding movement backward in time.

At any time step, the probability that an individual will reproduce is based on its proximity to either of the two peaks on the landscape and the current population size. Further, the probability that the individual will survive to the next time step is also based on the same criteria. In the discussion below, M (the number of peaks) = 2.

First, we define the landscape via a height function for each point x.

$$height\left(x,\hat{H},\;\hat{S,}\hat{X}\right)=\sum_{i}\;h_{i}e^{-\;{\left(\frac{x-x_{i}}{s_{i}}\right)}^{2}}$$

where $\hat{H}=\left(h_{1},h_{2},\ldots ,h_{M}\right)$ is a vector of peak heights, $\hat{S}=\left(s_{1},s_{2},\ldots ,s_{M}\right)$ is a vector of peak standard deviations, $\hat{X}=\left(x_{1},x_{2},\ldots ,x_{M}\right)$ is a vector of peak locations. When x is located at one of the peaks, height will be roughly equal to the peak height. Next, we define the probability that an individual will reproduce based on its location and the overall population n.

$$P_{repro}\left(x,n\right)=R_{repro}\left(n\right)\left[\alpha +\left(1-\alpha \right)\frac{height(x)}{1+height(x)}\right]$$

where R_repro_ is a scaling function based on the current population and α helps define a lower limit for the reproduction probability. Note that as height → ∞, P → R_repro_. Finally, we define the probability of survival to the next time step by scaling the probability of reproduction. Here, R_survive_ is a scaling function based on current population.


$$P_{survive}\left(x,n\right)=R_{survive}\left(n\right)P_{repro}\left(x,n\right)$$

## Results

**Fig 3:**
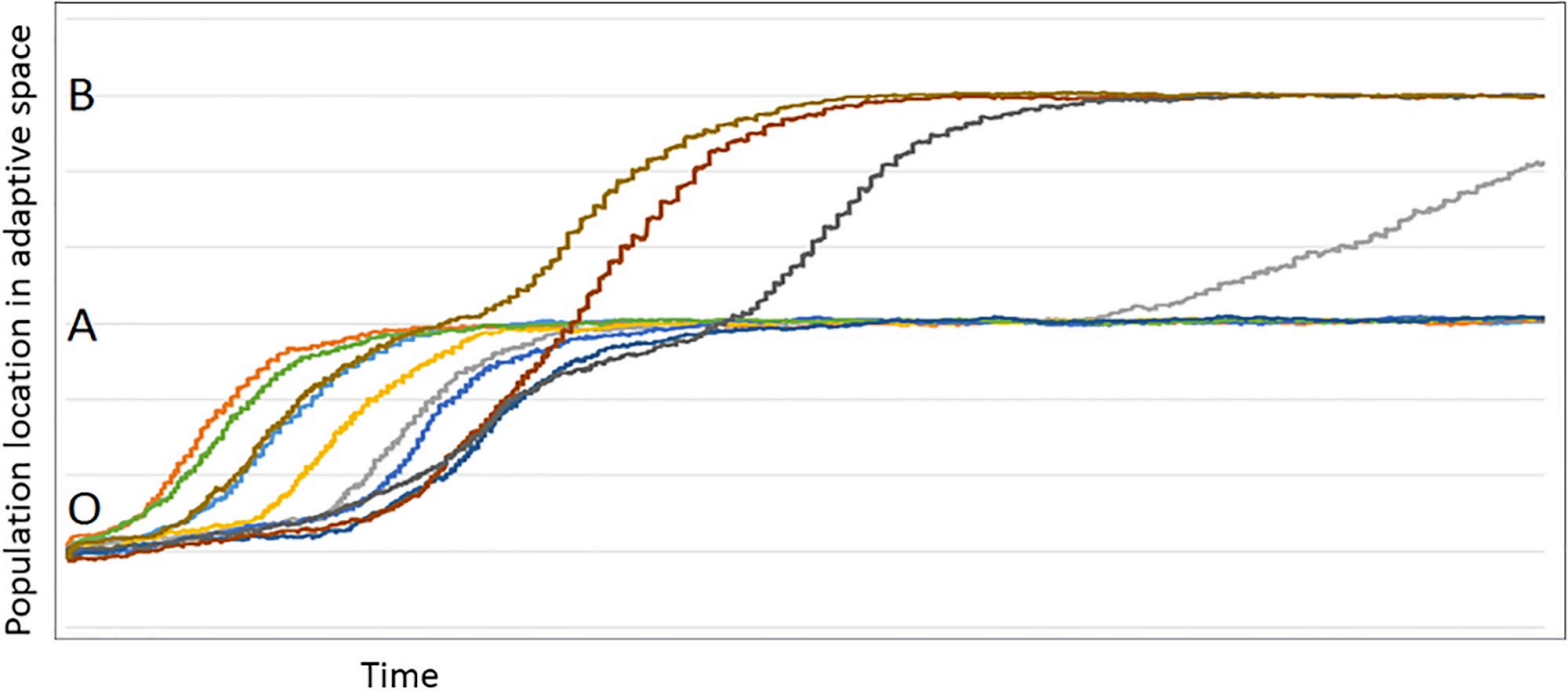
Results of the simulated fitness landscape model (n = 500 agents; 5,000 generations). A typical example of a cycle through the model. In this instance, the distant peak (B) is twice as far from the origin (O) as the near peak (A). The colored lines represent the populations at any given time, and each color is a different iteration (varying strength of B relative to A). The horizontal axis is time, and the vertical axis is the location of the population in adaptive space. For each iteration, the height of Peak B is increased, increasing its ‘attractiveness,’ starting equal to A and ending at B, having 20x the attractiveness of A. Most populations remain at the closer peak (A), and only when the ‘height’ of the more distant peak (B) is 1.5x or greater do the populations migrate through Peak A and into Peak B.

**Fig 4:**
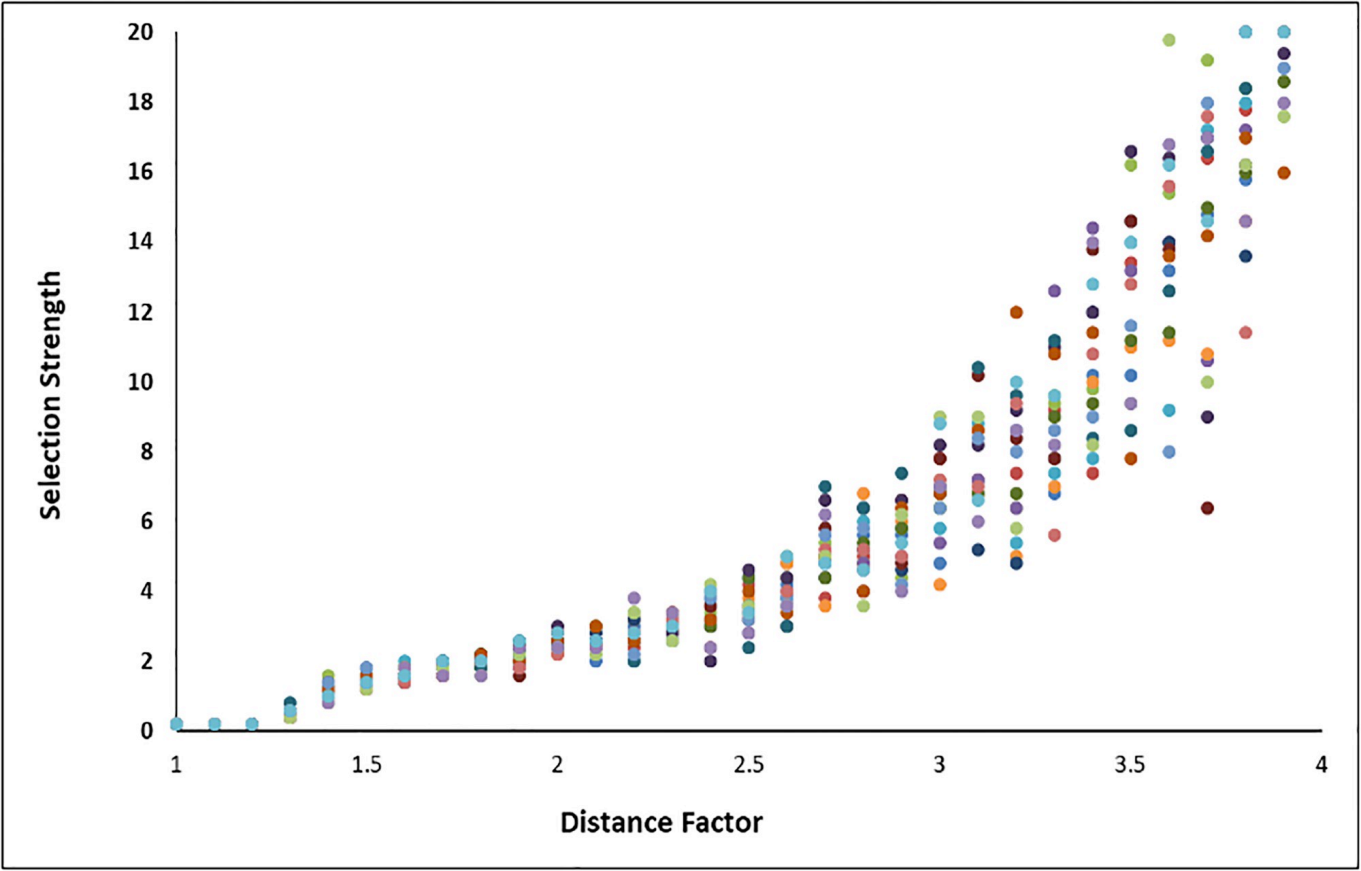
Height of the peak necessary to attract the population. The height of the far peak (Peak B) necessary to attract the population as that peak becomes more distant over multiple repeated iterations, is shown. Each iteration is represented by a different color. The more distant the far peak, the greater the height of that peak is necessary. Here, the distant peak starts at the same location as the near peak (X-axis value of 1) and increases. The X-value is the distance factor, so ‘2’ is twice as far as the near peak. The height of the peak necessary to attract the population is on the Y-axis. As the far peak recedes, the strength necessary increases asymptotically. For this analysis, whenever the population arrived within one standard deviation of the distant peak, the population was deemed to have ‘arrived’.

Fig 3 shows a fairly typical result. In all cases, the more distant peak was unlikely to be ‘found’ unless the strength of that peak was many times the near peak. In the simple case where the distant peak is twice as far, the strength of that peak must be as much as 1.5 to 2.5x that of the near peak to consistently be selected. For further cases, the distant peak must be even stronger (Fig 4).

Applying a random walk model demonstrates how problematic it is for natural selection to favor the more temporally distant adaptation. In most circumstances, the cost of moving off the short-term peak is too great for the population to migrate toward the distant peak (in a reasonable amount of time– 5000 generations), even when the value of the distant peak is mathematically greater (typically many times greater). The more distant the far peak, the greater its height must be to attract the population off the near peak. The pattern of the heights necessary shows an asymptotic pattern as the distance increases (Fig 4). We infer the mathematical relationship:$P_{2}=(h,t)=h{\left(\frac{v}{t}\right)}^{2}$. Where the *p*_*2*_ is the probability of the population moving off a near peak, *h* is the height of the more distant peak, *t* is the temporal distance from the near peak to the old peak, and *v* is the population variance (here held constant).

Our inference from the simulation results is that selection can more easily act upon near-future events than far. Also, the event in the future must be *dramatically* more beneficial for the organisms for them to pass over the near peak because of the cost of abandoning the advantages of that peak, even if it is smaller (e.g., 1.5–2.5x higher for only twice as far, but up to 20 times as high for peaks 3–4 times as far). These results are consistent with previous work identifying the difficulties of moving from low peaks to high peaks, an observation made by Wright [72] in his initial description of the fitness landscape. However, one important result of this model is that, with a sufficiently strong selective force, it is possible for natural selection to respond to temporally distant rewards.

### Model—Part 2: Uncertainty model

We argue that there is an important difference between development and behavior with regard to predictability. For many of the external domains in which organisms operate, the high number of interacting elements and the unpredictability of the results of small changes in initial conditions create high levels of uncertainty. Some of these systems behave chaotically [81–84]. Organisms must live in an external environment; modern analyses of climate systems emphasize the chaotic nature of these systems, and even the best and most sophisticated climate models can only predict weather effectively a few weeks into the future [85–87]. Similarly, ecological systems are modeled as chaotic [88–90]. The unpredictable nature of the external environment presents a significant problem for natural selection on future behavior because if a future event is to be selected for, it must, with some reliability, actually be ‘present’ in the future. Selecting for future events cannot happen if the future event does not occur predictably.

This contrasts with the patterns of developmental biology. Analyses of development have generally not found the same degree of non-linearity as seen in ecology and climate studies. Although there is some potential degree of uncertainty in the genetics of organisms due to the potential for epigenetic changes (i.e., altering gene expression) and random mutations to be benign, fitness-enhancing, or deleterious, it appears that, across the broad scale, development is consistent and predictable, with little evidence of a strong effect of chaos [91, 92], although some degree of chaos may play a role in the maintenance of genetically inherited deleterious diseases [93–96]. This likely arises because multiple mechanisms have evolved to ensure genetic stability, fidelity, and repair (e.g., canalization, DNA double helix, DNA mismatch repair (MMR) genes, DNA polymerases, etc.–[97, 98] that cannot apply in the external circumstances of an organism. This places an additional barrier on selection for temporally distant behavior, whereas this barrier does not apply to development. Any adaptation for a temporally distant event *exterior* to the organism may have to deal with the chaotic nature of the external environment.

### Uncertainty model

**Fig 5:**
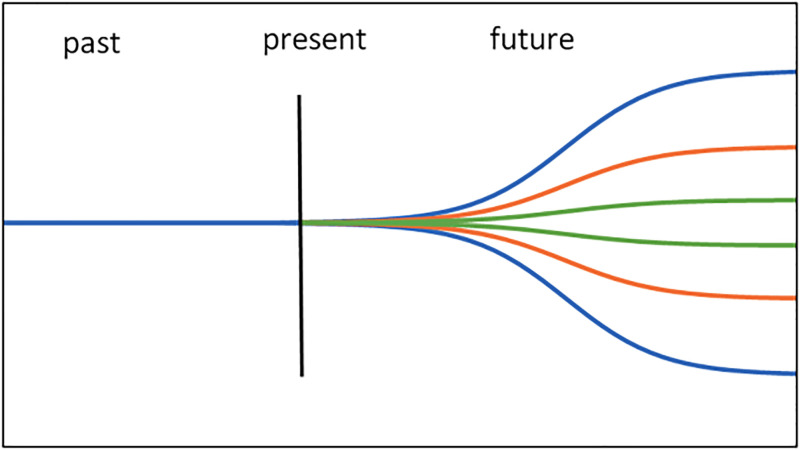
Model of potential outcomes over time. Here, the model of potential outcomes over time (the phase space) for any single parameter, using a bounded logistic model is derived from empirical analyses of predictive uncertainty in climatology, is visualized. In this figure, using time flows from left to right. Future outcomes (right) are more unpredictable the further they are from the present but ultimately are bounded. The different color curves represent varying degrees of predictive imprecision, which reflects varying values for *z* in the logistic function. The green curve is least affected by uncertainty, and the blue the most.

For evolutionary models, we regard the potential result space for any given parameter (the phase space) as being bounded because there are, practically speaking, limits to the potential parameters of any system in which animals might be making ‘decisions’ about development or behavior (Fig 5). To express the boundedness of the potential outcomes, we apply the relatively simple logistic function, such that the predictability of a future event becomes a factor of time to the event within the bounds of the domain. The logistic model is widely used to model predictive uncertainty, but we specifically borrow it from recent analyses of (in)accuracy in meteorological forecasting [85–87] because weather (e.g., short-term temperature, humidity, precipitation, etc.) and climate (long-term pattern of weather in a given area), present and future, are such strong factors in organismal survival.

### Methods—Part 2: Uncertainty model

For this model, we estimate predictability $P_{1}(t)=\frac{-1\;\left(z\right)}{1+\;e^{-k\left(t\right)}}$ where *t* is the time from the present to the future event, *z* is the boundedness relevant for the given parameter being predicted, and *k* is the steepness of the logistic curve (here held constant). Some types of domains with low predictabilities will have a very low *z*, whereas some, where the potential outcomes are more constrained, will have a high *z*. Natural selection will favor domains with more predictable outcomes, and in this formulation, a lower *z* (higher predictability) reflects a domain on which natural selection might more easily act on a future event. However, time is also a significant factor. Events in the near future are also more predictable [99], so we predict that natural selection is more able to act on events in the near term than the far. Note that we treat the distribution as uniform since we have no reason to predict any sort of normal probability distribution within the bounds of the uncertainty phase space.

**Fig 6:**
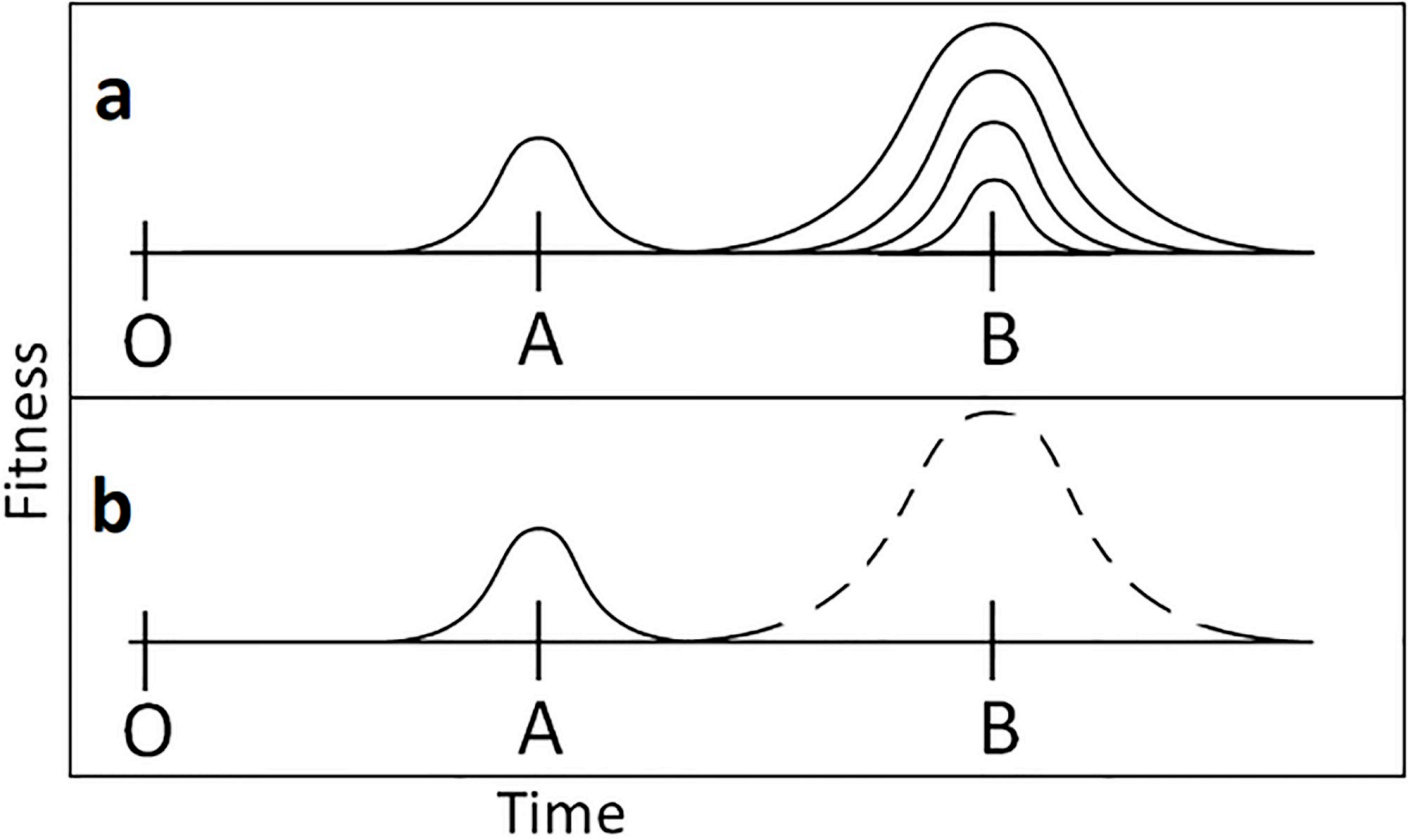
a and b–Models Employing Uncertainty. Here, visualizations of the two models employing uncertainty are represented using the fitness landscape. In the first model (upper), the height of the temporally distant peak is probabilistically attenuated according to the logistic function. In the second, the distant peak is variably absent, also following the probability of the logistic function.

We test the effects of uncertainty on selection for future events by applying this ‘uncertainty model’ to our first ‘distance model’ in two ways, both of which follow the general principle that a distant reward, such as cached food, can only act as a selective force if it is actually present in the future (Fig 6). It is important to note that these models *do not in any way simulate the factors driving the uncertainty* (such as the cognitive abilities of the organisms or the effects of chaos); rather, they model the potential cumulative effects of the predictive imprecision for organisms living and operating in an uncertain future environment.

For the first (Fig 6a), uncertainty is modeled as attenuating the height (strength) of the future peak (as might be expected, for example, with cached food whose value to a planning organism might be variably affected by weather or parasites). The effect of uncertainty here is modeled by applying the logistic model to the selective strength of the distant peak. To model this under the conditions of uncertainty, the height of the distant peak is treated as ‘uncertain.’ A probability function (as above) is generated that affects the ‘height’ (or selective strength) of the distant peak. The more distant the far peak, the more likely it is to have a reduced height, using the probability function. Under this model, a more distant peak may still have a strong selective force, but the probability of that selective force reliably appearing over many generations is reduced the further into the future it is. The effect of uncertainty is recalculated every generation, but each organism has its own probabilistic result based on the logistic function. We report results for the different ‘amounts’ of uncertainty, as reflected in the variable *z* in the above function.

For the second (Fig 6b), uncertainty is modeled as affecting the presence or absence of the distant peak. If the future reward is somehow eliminated (for example, if the cached food is stolen), then that distant peak is eliminated altogether. For this, the probabilistic function determines whether the distant peak is absent or present. This is expected to be a less conservative test of the effect of uncertainty on the two-peak model. As mentioned above, the probability function is recalculated every generation, but each individual ‘organism’ has an individual result of that probability calculation. As above, we report results for the varying degrees of uncertainty introduced into the system, iteratively increasing the variable *z* in the logistic function the further in the ‘future’ the distant peak lies.

## Results

**Fig 7:**
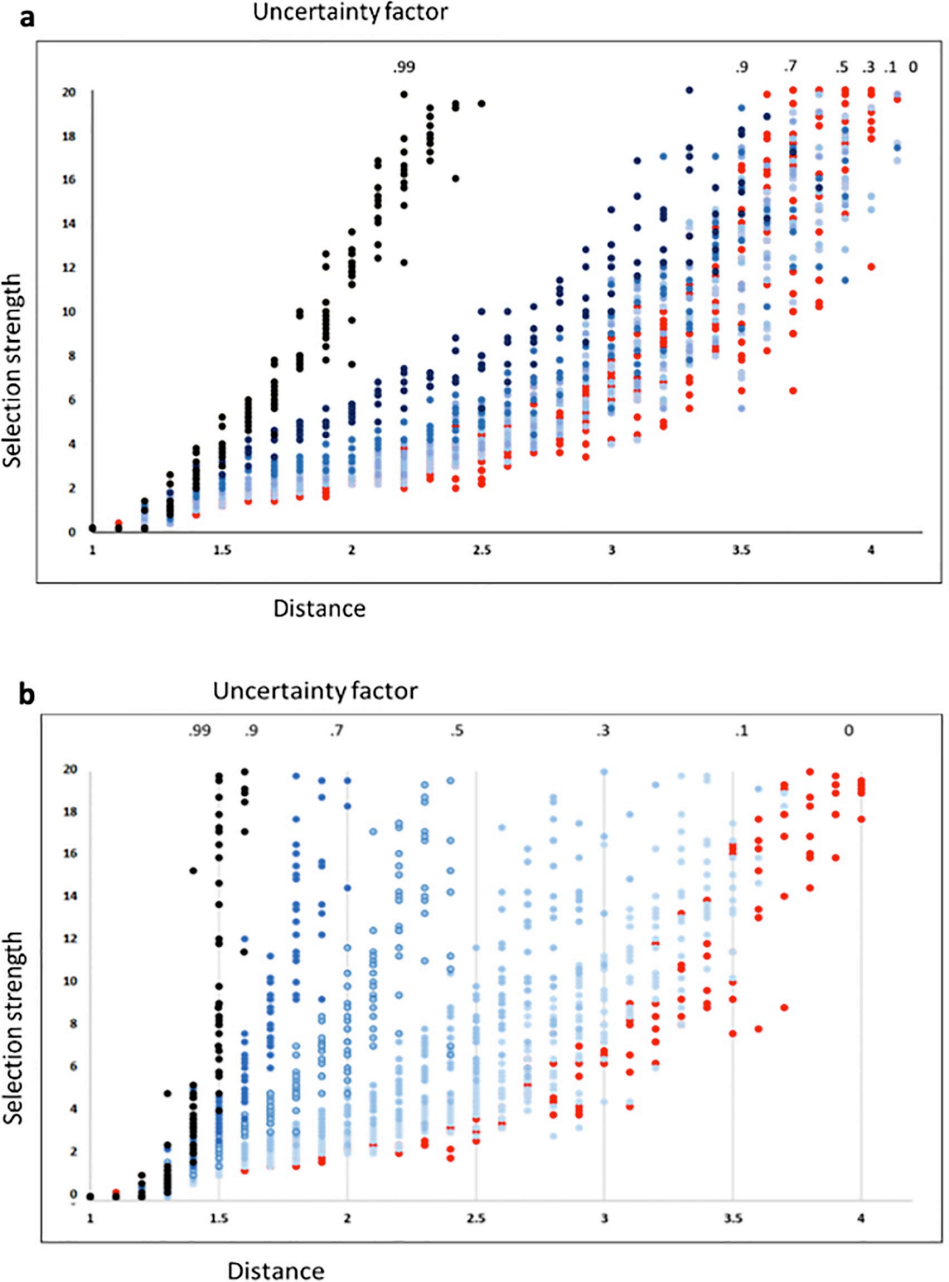
a and b–Effect of Uncertainty on Selection for Future Events. Here, the effect of uncertainty on selection for events in the future using models in which the height of the distant peak is affected (a), and the presence/absence of the peak is affected (b), are shown. The left axis is as in Fig 4 (the strength of the distant peak necessary to reach the destination peak). The lower axis (as in Fig 4) is the distance of that peak. That number indicates how far into the future a peak could be reached for a given influence of uncertainty (upper number, corresponding to *z* in the formula in the text). A distribution that fails to reach horizontally across the graph indicates that, even with maximum selective pressure (20x), the population could not reach a peak beyond the number indicated on the lower horizontal axis. So, for example, in Fig 7b, the uncertainty (*z*) factor of .5 prevented the population from finding a peak beyond 2.4 times the original distance from the origin (O) to Peak 1, irrespective of the selection strength for that peak.

The results of the two uncertainty models are seen in Table 1 and Fig 7. The first model produced a result in which any ‘future’ peak became increasingly difficult to reach as uncertainty increased. In the second model, in which the ‘future’ peak intermittently but probabilistically disappeared due to natural selection, the result was more widely dispersed, with results for strong ‘uncertainty’ factors even more dramatic. Here, any peak just over twice as far into the future was completely unreachable (no matter how high the peak) by the population for an uncertainty factor of 0.5. For both models, adding uncertainty to the two-peak fitness landscape model makes selection for any future event very difficult to achieve. The strength of the *z* factor does play a role (as in Fig 6a and 6b), but even adding a modest degree of uncertainty to the distant peak has a clear effect on the ability of natural selection to ‘find’ that peak and select for it. (Note that in our simulations, beyond a distance of 4, the algorithm became computationally cumbersome).

**Table 1:** The effects of uncertainty on the adaptive peak model after multiple iterations of each model, for populations of 500 (5000 generations, 100 cycles of selection strength increase in peak 2 height, by factors of .2, for 50 iterations of increasing distance for peak 2, repeated 50x). In this table, the column headers represent the degree of uncertainty, from 0 (no uncertainty) to .99 (maximum uncertainty). The number in the cells are the average distances achievable under each level of uncertainty, after increasing selection strength for Peak B up to 20x the strength of Peak 1. With no uncertainty, the populations could reach peaks 4x as far as Peak 1, but this reduces to only 1.4 as far under the maximum uncertainty, using Model B.

Uncertainty factor →	0	0.1	0.3	0.5	0.7	0.9	0.99
**model a**	4.0 ± 0.5	3.9 ± 0.8	3.9 ± 1.3	3.8 ± 1.9	3.7 ± 1.4	3.6 ± 1.1	2.3 ± 1.1
**model b**	4.0 ± 0.5	3.5 ± 1.1	2.9 ± 1.1	2.3 ± 0.9	1.9 ± 0.8	1.5 ± 0.6	1.4 ± 0.4

We interpret this result as supporting the idea that uncertainty in a domain makes selection for events in that future difficult. The uncertainty of the future in a chaotic or stochastic system creates significant obstacles for organisms ‘planning’ for that future since that event may not appear, or its positive effect (reward) may be attenuated.

## Discussion

This study investigated how natural selection, time, and unknown future risks and rewards interact to shape an organism’s (specifically animal) behavioral phenotype. The models tested hypotheses about the relationship between an adaptive response and temporal distance into the future. The results show that the temporal distance into the future is the most important factor driving the overall pattern. The adaptive peak model (Model part 1) indicates that natural selection will not promote individuals toward the more advantageous adaptive peaks located in the future if it requires passing over a lesser reward closer to the present unless the future peak is several times higher (e.g., 1.5–2.5x higher for only twice as far, but up to 20 times as high for peaks 3–4 times as far). Simply put, the *cost* of descending off the near peak is typically too great for a given population (Figs 1–4). Here, we propose an ultimate (i.e., evolutionary) causal explanation for the selection of temporal discounting across taxa. We propose that preference for a short-term reward (e.g., heightened impulsivity) often is less than optimal and likely is the product of constraints imposed on natural selection with respect to predicting events in a temporal framework in the context of future uncertainty.

However, variations in the timing of developmental patterns across taxa that unfold over years or even decades indicate that natural selection does act on anatomical and physiological phenotypes in the future in many circumstances. Yet, the results of Model Part 2, in which uncertainty is a factor, imply that, for behavioral phenotypes, the further into the future an event is, the less reliable the prediction from past events are and the less likely it is to be ‘visible’ to natural selection to act upon, which in turn dramatically reduces and weakens selective pressure. Even strong selective pressure cannot move the population towards the more distant peak if it is not visible to selection. As a result, natural selection is often constrained across most taxa to promote optimal long-term decision-making behavioral strategies.

When examining the interaction of time and natural selection processes, one key issue is understanding the way in which a temporally distant event can produce selective pressure. For a future event to produce a selective pressure, it must actually exist in the temporal dimension for any given individual. In other words, it must reliably produce an evolutionary benefit to an individual organism. If a western scrub-jay caches worms for future consumption, it must, in some sense, ‘know’ that the food will be there when retrieved. The extent to which an orb-weaver spider (*Argiope savignyi*) reclaiming cached food ‘knows’ anything is certainly debatable, but the behavior of caching and storing food is only selected for if the food is present in the future when needed [100, 101]. A critical aspect of our models’ results is that the reliability of the future event is likely to play an essential role in the selection of a behavior that relies on that event. If a scrub-jay caches food and the food is stolen, or it forgets the cache location, the result is a maladaptive behavior that would likely be negatively selected against. But a cache that can be reliably accessed might well enable the survival of an animal during a lean winter. Under natural conditions, uncertainty and risk are likely to exert considerable constraints on driving optimal decisions. These external conditions, such as weather or the behavior of other animals, likely increase uncertainty [102]. For cognitively advanced animals that can discern degrees of uncertainty for future events, it appears that assessment of reduced risk mitigates time preference [68, 69]. Further, in experimental contexts, some behavioral domains generate more time preferences than others, suggesting that different domains have different degrees of predictability [103].

Although our model applies to any behavior, including those other than foraging (e.g., mate choice, social relationships, etc.), our results are consistent with predictions derived from risk-sensitive foraging theory [104–106] in which the decision to choose a riskier strategy is partly driven by predictability [5, 106] and time preference [4, 6], as well as the relative value of the riskier reward [107–109], framed in the context of the present state of the animal [e.g., closer to starvation, solitary versus in a group, etc.; 8, 104; 106, 107, 110, 111]. Experimental results have provided mixed support for various models [50, 108, 112]. However, one interesting recent conclusion is that temporal information may be a valuable factor in behavior when available [7, 113]. Our results do not specifically address any one behavioral dimension, but they do explain the way in which future benefits are mitigated by the mathematical cost of selection operating on a temporally distant reward.

### Implications for human evolution

Examples of short-term cognitive biases in human behavior are well-established and diverse, particularly in domains ranging from discounting the long-term impacts of climate change, negatively impacting one’s health for a reproductive payoff (e.g., risk-taking), and irrational long-term economic decision-making [e.g., lack of planning for retirement; 6, 37, 39, 40, 114].

Our results help to illustrate why temporal discounting among hominids was likely favored. The preference for short-term reward has been documented in great apes, monkeys, and other closely related animals and would have likely been conserved in hominins, including *Homo sapiens* [45, 58, 68, 103, 115–118]. The first evidence of the emergence of the genus *Homo was* ~2.8 million years ago [119]. During this time, hominins evolved and speciated in the terrestrial environments of Africa and later spread throughout Eurasia [120], where they faced adaptive challenges related to a nomadic, scavenger, and later, foraging subsistence strategy [121]. For ~98% of *Homo sapiens*’ ~300,000-year existence [122], well before the advent of sophisticated food preservation techniques and agriculture, predicting future circumstances beyond a few weeks would have been extremely difficult and unlikely in the environment of evolutionary adaptedness for ancient small-scale nomadic foragers [123]. Selection pressures in the absence of information on the future would have predictably operated strongly to forge psychological adaptations that preferentially respond to immediate short-term rewards [68, 113, 124], such as food resources and mating opportunities [with respect to males in particular; 125, 126].

*Homo sapiens* first transitioned to agriculture ~11,000 years ago BCE [127], and by ~6,000 years ago, it was widely practiced globally [128]. Such a dramatic cultural change in subsistence strategy profoundly altered the ecological and social landscapes in which *Homo sapiens* had evolved previously. With the co-emergence of agriculture, food surpluses, advanced preservation techniques, and advances in domestication technology, humans could make more reliable long-term (seasonal, annual) investments in the future [127, 128]. In some ways, these technological and social advances perform the same function as canalization, DNA polymerase enzymes, and MMR genes–to buffer the individual against unpredictability, which may help explain the dramatic pace and scale of the spread of agriculture independently worldwide.

The rate at which human civilization advanced from agrarian to industrialized to post-industrialized societies far outpaced the rate at which polygenic and *complex* behavioral phenotypes could possibly significantly adapt, as evidenced by humans’ persistent and even increased rates of temporal discounting observed today among urban, resource-rich populations [129]. These findings raise intriguing questions about whether temporal discounting may be exaggerated in dense urban populations due to evolutionary mismatch [130].

The results from our models highlight why the persistent phenomenon of temporal discounting is still so difficult to overcome, even when the long-term benefit of delaying an immediate reward is overt and well-described to human participants [for review see 131]. Consistent with the findings presented here, we suspect that early *Homo sapiens* and their hominid predecessors likely did not experience significant fitness-related benefits from investing in future events.

## Conclusion

These findings provide a deeper conceptual framework to appreciate why human and non-human animals can reliably produce efficient developmental and anatomical changes over their lifetimes, yet paradoxically, so often fail to produce more optimal behavioral adaptations across temporal dimensions [9]. Our model indicates that natural selection is constrained to overcome short-term adaptive peaks due to the influence of uncertainty. As a result, the allure of short-term benefits in an unpredictable world is often too much to overcome for most species to produce more optimal behavioral phenotypes across temporal dimensions. The findings presented here provide a broad application to the Life Sciences, with particular interest to Life History Theorists, Evolutionary Psychologists, Behavioral Ecologists, Evolutionary Developmental Biologists, and Ethologists.
